# Simple and Rapid Determination of Ferulic Acid Levels in Food and Cosmetic Samples Using Paper-Based Platforms

**DOI:** 10.3390/s131013039

**Published:** 2013-09-26

**Authors:** Prinjaporn Tee-ngam, Namthip Nunant, Poomrat Rattanarat, Weena Siangproh, Orawon Chailapakul

**Affiliations:** 1 Petrochemistry and Polymer Science, Faculty of Science, Chulalongkorn University, Patumwan, Bangkok 10330, Thailand; E-Mail: jui_jitzu14@hotmail.com; 2 Electrochemistry and Optical Spectroscopy Research Unit, Department of Chemistry, Faculty of science, Chulalongkorn University, Patumwan, Bangkok 10330, Thailand; E-Mails: nongpang_2527@hotmail.com (N.N.); r.poomrat@hotmail.com (P.R.); 3 Department of Chemistry, Faculty of Science, Srinakharinwirot University, Sukhumvit 23, Wattanna, Bangkok, 10110, Thailand; 4 National Center of Excellence for Petroleum, Petrochemicals, and Advanced Materials, Chulalongkorn University, Bangkok 10330, Thailand

**Keywords:** ferulic acid, paper-based platforms, electrochemical detection, colorimetric detection, TLC separation

## Abstract

Ferulic acid is an important phenolic antioxidant found in or added to diet supplements, beverages, and cosmetic creams. Two designs of paper-based platforms for the fast, simple and inexpensive evaluation of ferulic acid contents in food and pharmaceutical cosmetics were evaluated. The first, a paper-based electrochemical device, was developed for ferulic acid detection in uncomplicated matrix samples and was created by the photolithographic method. The second, a paper-based colorimetric device was preceded by thin layer chromatography (TLC) for the separation and detection of ferulic acid in complex samples using a silica plate stationary phase and an 85:15:1 (v/v/v) chloroform: methanol: formic acid mobile phase. After separation, ferulic acid containing section of the TLC plate was attached onto the patterned paper containing the colorimetric reagent and eluted with ethanol. The resulting color change was photographed and quantitatively converted to intensity. Under the optimal conditions, the limit of detection of ferulic acid was found to be 1 ppm and 7 ppm (S/N = 3) for first and second designs, respectively, with good agreement with the standard HPLC-UV detection method. Therefore, these methods can be used for the simple, rapid, inexpensive and sensitive quantification of ferulic acid in a variety of samples.

## 1. Introduction

Antioxidants are compounds required by most organisms, where they prevent the oxidative damage caused by free radicals, including in humans where they otherwise may contribute to various diseases, such as cancer, cardiovascular diseases and cataracts [1]. Interest in natural antioxidants has increased significantly in the past few years. The antioxidant activities of plants have been attributed mostly to their phenolic content, which is one class of natural antioxidant. Therefore, plants containing a high-level of phenolic acids have a high potential importance as a source of natural antioxidants. Ferulic acid (4-hydroxy-3-methoxycinnamic acid) is one of many ubiquitous phenolic acids in the plant kingdom. A high amount of ferulic acid is found in corn (2510 mg/100 g), wheat (391 mg/100 g) and flax (18 mg/100 g) [2]. Ferulic acid is more easily absorbed into the body and stays in the blood longer than any other antioxidant, even longer than vitamin C. Because of these features, ferulic acid is considered to be a superior antioxidant and accordingly it is widely used in health foods and nutrition restoratives. In addition, ferulic acid has been touted as an anti-microbial, anti-inflammatory, anti-arrhythmic and anti-thrombosis pharmacological agent [1]. Ferulic acid is also used in a wide range of cosmetics, such as skin lighteners, moisturizers and sunscreens, because it has the ability to protect the skin from ultraviolet radiation. Ferulic acid also reduces nerve cell damage and may help to repair damaged cells. Furthermore, it is a popular sports supplement because, as an antioxidant, it can neutralize free radicals in muscle tissue and so can alleviate muscle soreness, loss of endurance and muscle fatigue. In addition, ferulic acid was found to be able to alleviate oxidative stress in diabetic rats and to lower their blood glucose levels [3], as well as to reverse the damage to the organs in diabetic rats. Thus, the antioxidant properties of ferulic acid might be beneficial for diabetics. In addition, ferulic acid has been shown to be effective in treating menopausal hot flashes, which may be caused by inflammation and oxidation of tissues, and also has immune stimulant properties [4], and has also been shown to suppress many types of cancer, such as lung, liver and digestive tract cancers [5,6]. In rats, ferulic acid is reported to increase HDL levels, and decrease the total cholesterol and triglyceride levels [7], which could potentially suggest a role in regulating some cardiovascular diseases.

Currently, the main quantitative techniques for ferulic acid detection are high-performance liquid chromatography (HPLC) [8–10], thin-layer chromatography (TLC) [11–13], capillary tube electrophoresis [14–16], and spectrophotometry [17–19]. Wu *et al.* [20] established a HPLC method for the simultaneous determination of hesperidin (HE), ferulic acid (FA), cinnamic acid (CA) and cinnamaldehyde (CAD) in a Chinese tonic wine.The limit of detection (LOD) for ferulic acid was found to be 0.075 g/mL. Luo *et al.* [21] developed a method for the determination of ferulic acid based on the chemiluminescence reaction. The detection limit for ferulic acid was 8.7 × 10^−9^ mol·L^−1^. However, these assays require laborious sample preparation steps, high equipment costs and a significant amount of labor and analytical resources, which can potentially cause substantial delays in obtaining the results. Moreover, electrochemical detection has been also used for quantitation of ferulic acid [22–24]. Electrochemistry is an attractive and alternative technique due to its high speed, portability, high sensitivity, and selectivity. Ya-Ping Ding *et al.* [23] investigated the electrochemical behaviors of ferulic acid using the glassy carbon electrode modified with a multi-walled carbon nanotube. The limit of detection was estimated to be 1 × 10^−7^ M. Moreover, Jia *et al.* [24] reported a method for the determination of ferulic acid by didodecyldimethylammonium bromide (DDAB)/Nafion composite film-modified carbon paste electrode. The detection limit of ferulic acid was found to be 3.9 × 10^−7^ M. The limits of detection (LOD) in these methods are typically low enough to detect ferulic acid in real samples. From the previous work with electrochemical sensor, the modified electrode is required and they have limitation due to the tedious preparation steps. Thus, the development of a new method for the rapid and inexpensivequantification of ferulic acid is very important.

Paper-based analytical devices have the potential to be good alternative analytical devices for healthcare related applications because they are portable, easy to use, require only a small volume of sample and provide a rapid analysis [25–27]. For forming the patterning channels of hydrophilic surfaces on filter paper, there are several methods available, including photolithography, wax printing and wax screen-printing. Photolithography was first used as a simple method using negative photoresist to create a small hydrophilic channel on a millimeter scale on chromatography paper [25]. Wax screen-printing can also fabricate hydrophobic patterned channels on paper [28], and has the advantage of being more environmentally user-friendly, inexpensive and simpler than photoresist. Recently, paper-based analytical devices coupled to colorimetric or electrochemical detection were successfully fabricated for several different application areas [28–34], and this alternative type of detection method has the benefits of simplicity, speed, low cost, and portability. However, because of the lack of analyte selectivity of both these detection modes, an extensive sample preparation step was required. Additionally, there are information about the combination of sample preparation and paper-based analytical devices [35–37]. Recent works using paper based devices were reported successfully analyzing for biomarkers for liver functions [38], total protein in blood [39] and for bacterial DNA extraction from viscous samples [40]. Therefore, efforts to extend the developed methodology and/or to create new sample preparation devices for ferulic acid detection have been challenging. To reach these goals, two platforms of a (i) paper-based analytical electrochemical device and (ii) TLC coupled with a paper-based colorimetric device were designed and developed for the rapid detection of ferulic acid in simple and complex matrices.

The ferulic acid content in simple matrix samples (in this study, corn milk and corn cider) was directly determined electrochemically using the paper-based screen-printed electrode. For the complicated matrix samples (in this study, cosmetic creams), the use of TLC separation was first performed followed by detection with the colorimetric paper device, so as to minimize the interference effect upon the colorimetric detection of ferulic acid. The amount of ferulic acid was determined by monitoring the oxidative current and the color intensity at the zone of detection. Strong analytical figures with LOD in the low ppm range, good sensitivity, excellent response precision and stability were observed by both the electrochemical and colorimetric detection systems. Therefore, this innovative concept could contribute to the development of a practical, rapid, highly sensitive and accurate method for assaying ferulic acid levels in a variety of food and cosmetic samples.

## 2. Experimental Section

### 2.1. Apparatus

Cyclic voltammetry (CV) and differential pulse voltammetry (DPV) were performed using a potentiostat (Autolab PGSTAT 30, Netherlands). For voltammetric experiments, CV was performed at a scan rate of 100 mV/s and the potential was scanned from −0.2 V to 0.8 V *vs.* Ag/AgCl. DPV was performed at a pulse amplitude of 50 mV and a step potential of 7 mV in the potential range from 0 to 1.0 V *vs.* Ag/AgCl. For the colorimetric method, a digital camera (EOS 1000D, Canon, Japan) was used to capture the picture and then the picture was quantified for the mean color intensity using Adobe Photoshop (Adobe Systems, Inc., San Jose, CA, USA) [41].

### 2.2. Reagents and Solutions

A negative photoresist SU-8 3025 and developer were purchased from MicroChem Corp. (Newton, MA, USA). Carbon ink (Electrodag PF-407C) and Ag/AgCl ink containing Ag:AgCl ratio of 80:20 (Electrodag 7019) were purchased from Acheson (Ontario, CA, USA). Ferulic acid was purchased from Fluka (Buchs, Switzerland) and was prepared immediately before use. A 0.1 M acetate buffer, which served as a supporting electrolyte, was prepared from sodium acetate trihydrate and concentrated acetic acid (100%), which were obtained from Fluka (Buchs, Switzerland) and Merck (Darmstadt, Germany), respectively. TLC plates (silica gel 60 F_254_) were obtained from Merck (Darmstadt, Germany). The Folin-Ciocalteau reagent was acquired from Carlo Erba (Milan, Italy). Sodium carbonate, chloroform, formic acid and methanol were obtained from Merck (Darmstadt, Germany). All reagents were analytical grade. The 18 MΩ/cm resistance water, obtained from a Millipore Milli-Q purification system, was used throughout these experiments.

### 2.3. Fabrication of the Paper-Based Electrochemical Device Using Photolithography

Photolithography was used to fabricate the device (Figure S1) as described previously [25], using a spin coater (G3P-8, Speciality Coating Systems, Inc., Indianapolis, IN, USA) to coat 4 g of SU-8 negative photoresist onto the filter paper (No. 1, 100 cm diameter, Whatman), which was subsequently baked at 95 °C for 10 minafterwards, the photomask, which was designed with Adobe Illustrator software (Adobe Systems, Inc., San Jose, CA, USA) and fabricated by Chaiyaboon Co. (Bangkok, Thailand), was placed onto the SU-8-covered paper, irradiated with ultraviolet (UV) light (Intelli-ray 400, Uvitron International, Inc., West Springfield, MA, USA) for 10 s, and then baked at 95 °C for 10 min. Next, the unpolymerized photoresist layer was removed from the paper by soaking it in SU-8 developer for 3 min and then rinsed with isopropanol. Finally, the patterned paper was dried in a hood at room temperature.

**Figure 1.**
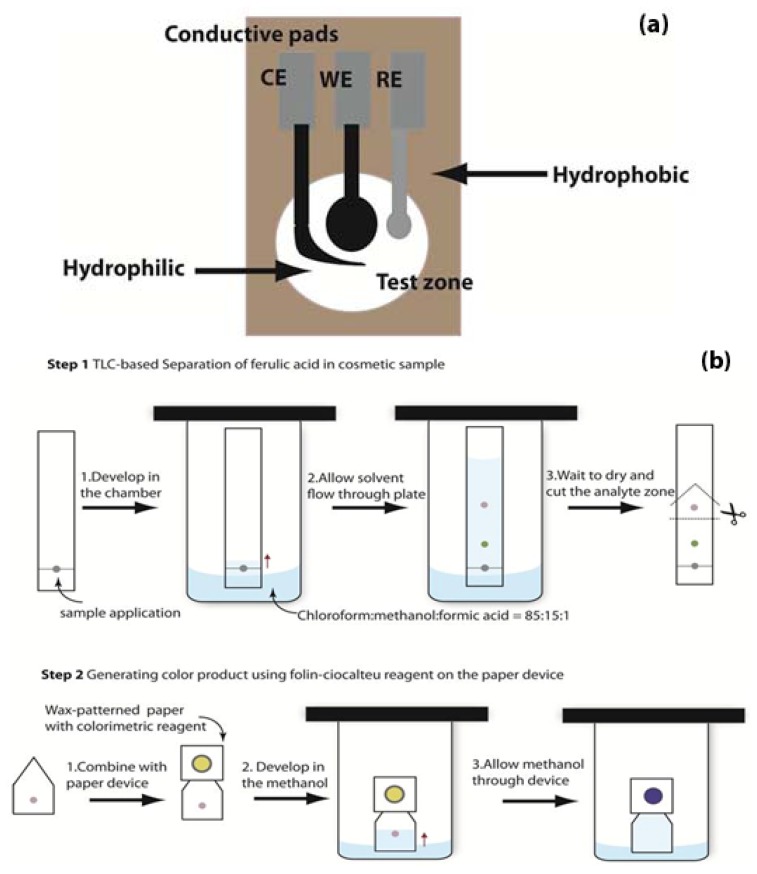
Schematic diagram of the (**a**) paper-based electrochemical device consisting of the electrochemical working electrode (WE), reference electrode (RE) and counter electrode (CE), and the (**b**) separation and quantitative analysis of ferulic acid by coupling TLC with the paper-based device.

For the electrode preparation, the screen-printed electrodes were prepared in-house [28]. Carbon ink was used for the working electrode (WE) and the counter electrode (CE); Ag/AgCl chloride ink was used for the reference electrode (RE) and conductive pads. All electrodes were screened on patterned paper and then were cured in the oven at 65 °C for 30 min. The paper-based device designed in this study for electrochemical detection is illustrated in Figure 1a.

### 2.4. Separation and Quantitative Analysis of Ferulic Acid by TLC Coupled with Paper-Based Colorimetric Platform

The wax screen-printing method was used to fabricate the patterned paper (Figure S2) as previously described [42]. The filter paper was positioned on the hot plate at 100 °C, and then solid wax was placed on the block screen. After the wax was melted, it was printed through the perforated screen and allowed to absorb into the paper. The patterned paper was ready to use after the wax was cooled to room temperature.

Ferulic acid was determined colorimetrically on the paper-based device using the Folin-Ciocalteau reagent [43,44]. 2 μL of Folin-Ciocalteau reagent was applied to the colorimetric test zone. Afterward, 2.5 μL of 10% (w/v) sodium carbonate was dropped onto this area and the paper-based device was allowed to dry at room temperature.

Figure 1b shows the conceptual separation and detection of ferulic acid using TLC coupled with the paper-based colorimetric device. For the separation of ferulic acid by TLC, the TLC plate was cut into strips (2 cm × 15 cm). 2 μL of 50 ppm Standard ferulic acid and ascorbic acid were applied on the plate and developed with a 85:15:1 (v/v/v) chloroform: methanol: formic acid mobile phase in a pre-saturated chromatographic chamber [11]. After separation (30 min), the TLC area containing the ferulic acid was cut and attached onto the paper-based colorimetric device. 5 mL of Ethanol was used to drive the ferulic acid onto the paper to react with the reagent on the paper-based device. The color change was completely occurred at ambient temperature for 15 min. The color of the test zone was captured by digital camera and analyzed for intensity using Adobe Photoshop [41].

### 2.5. Sample Preparation

Corn milk solution was prepared by weighing 5 g of commercially available corn milk and heating it at 90 °C in 50 mL of Milli-Q water. The samples were then filtered and used for analysis. For corn cider, the sample was used without any further preparation. These samples (as model simple matrix samples) were diluted once prior to ferulic acid determination by electrochemical detection. For colorimetric detection, the three samples of cosmetic serums (as model complex matrix samples) were used without any preparation. The major ingredients of these serums are L-ascorbic acid and ferulic acid which used to extend the life and effectiveness of L-ascorbic acid.

## 3. Results and Discussion

### 3.1. Paper-Based Electrochemical Device for the Direct Detection of Ferulic Acid

#### 3.1.1. Electrochemical Behavior of Ferulic Acid

**Figure 2.**
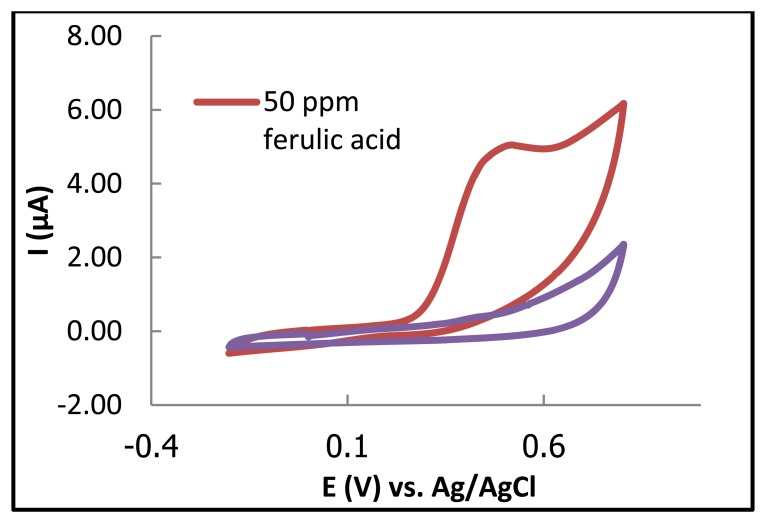
Cyclic voltammogram of 50 ppm ferulic acid in 0.1 M acetate buffer solution (pH 5). Scan rate 100 mV/s, electrode area 0.8 cm^2^. Voltammograms shown are representative of at least five independent repetitions.

First, CV was used to investigate the electrochemistry of ferulic acid on the paper-based electrode. Sodium acetate (0.1 M) buffer, pH 5.0, was used as the supporting electrolyte because it provides a well-defined peak at the carbon electrode [23]. Ferulic acid (50 ppm) exhibited a well-defined oxidation peak during the scan of the potential toward the positive direction at the carbon electrode at approximately 0.4 V *vs.* Ag/AgCl (Figure 2). The precision between electrode and electrode was examined from a series of six repetitive measurements of a solution containing 50 ppm ferulic acid. Reproducible signals were obtained with relative standard deviations (%R.S.D.) of 5% for the peak current. Such good precision indicates negligible surface reproducibility of the electrode preparation.

These results indicated that the paper-based carbon electrode offers great sufficient sensitivity for ferulic acid detection for use in electrochemical analysis.

#### 3.1.2. Effect of pH

**Figure 3.**
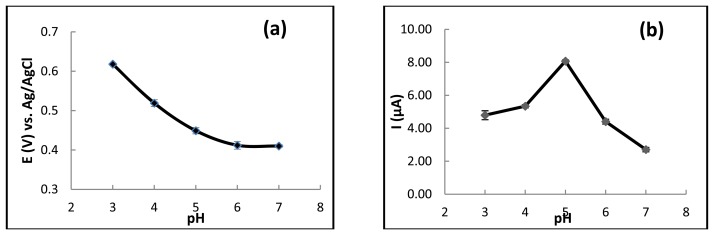
The influence of the electrolyte pH on the (**a**) oxidation peak potential, and the (**b**)oxidation peak current. Other conditions are the same as in Figure 2. Data are shown as the mean ± 1 SD and are derived from 3 independent repeats.

In any electrochemical detection method, the supporting electrolyte pH has a significant impact on the ionization and redox reaction of each analyte. Therefore, optimization of the supporting electrolyte pH was performed for the electrochemical detection of ferulic acid. The effect of pH (0.1 M acetate buffer) on the peak potential and oxidation current were examined in the pH range of 3.0–7.0. The relationship between the pH value and the oxidation peak potential from cyclic voltammogram (Figure 3a) revealed that ferulic acid (50 ppm) is easily oxidized as the pH increases from pH 3.0 to pH 6.0 because the potentials shifted negatively at the higher pH. Moreover, the relationship between the oxidation peak currents and pH was investigated. As shown in Figure 3b, it is evident that the highest current signal using carbon electrodes was obtained at pH 5. Accordingly, pH 5.0 was selected as the most suitable for all subsequent work.

#### 3.1.3. Effect of the Scan Rate

The influence of the scan rate was investigated in order to confirm the adsorption of ferulic acid on the carbon electrode surface by performing the CV over the potential range from 50 to 500 mV/s. The oxidation peak currents increased linearly as the square root of the scan rate increased as shown in Figure S3. Hence, this result confirmed that the reaction was controlled by diffusion process and that ferulic acid is slightly adsorbed on the electrode surface.

#### 3.1.4. Effect of Differential Pulse Voltammetric Parameters

**Figure 4.**
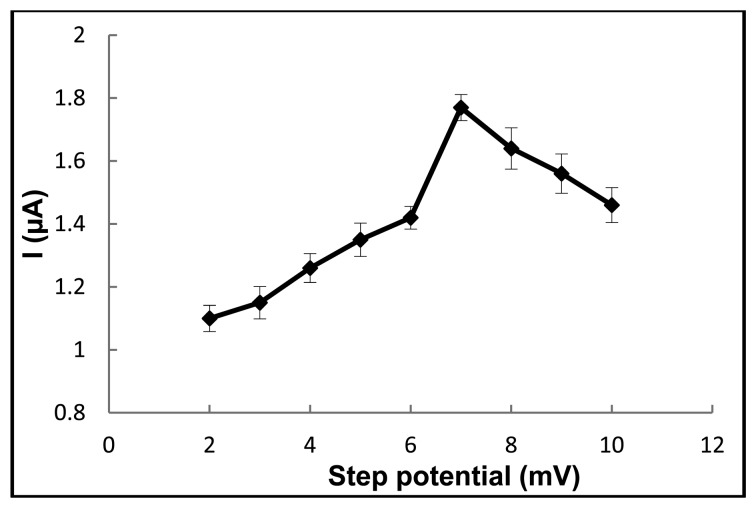
The influence of the step potential on the ferulic acid (50 ppm) oxidation peak current.Data are shown as the mean ± 1 SD and are derived from 3 independent repeats.

DPV is the technique typically used to achieve a lower detection limit than CV, and so DPV on this paper device was tested for ferulic acid detection (50 ppm). The differential pulse voltammogram of ferulic acid was clearly observed (Figure S4). To obtain the optimal conditions for the quantitative analysis of ferulic acid, the effects of the pulse amplitude and step potential on the peak current were examined. Initially the pulse amplitude was varied from 50 to 250 mV, where the peak currents of ferulic acid increased as the pulse amplitude increased, although a broadening of the peaks was also observed. Therefore, a pulse amplitude of 50 mV was chosen as a trade-off between the peak height and peak broadening. Then, the effect of the step potential was evaluated over the range of 2–10 mV, where the optimal value was found to be 7 mV because the increment in the peak intensity slightly decreased above 7 mV (Figure 4).

#### 3.1.5. Analytical Performances for Electrochemical Detection of Ferulic Acid

**Figure 5.**
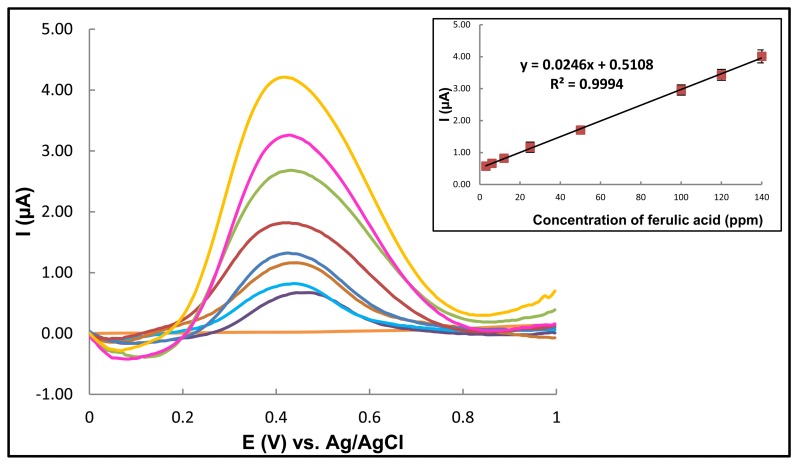
Differential pulse voltammograms of ferulic acid (3, 6, 12, 25, 50, 100, 120 and 140 ppm) in 0.1 M sodium acetate buffer solution (pH 5, measured under the optimal experimental conditions (see text).Data shown are representative of that seen in 3 independent repeats, or (insert) are shown as the mean ± 1 SD derived from the same independent repeats.

The ferulic acid solutions were investigated by DPV under optimal conditions (0.1 M acetate buffer pH 5.0, 50 mV pulse amplitude, 7 mV strip potential, and 100 mV/s scan rate). Defined peaks with currents proportional to the ferulic acid concentration in the applied sample were observed as shown in Figure 5. The oxidation peak current of ferulic acid showed a linear relationship with its concentration in the range of 3–140 ppm (correlation coefficient of R^2^ = 0.9994), with a sensitivity of 0.0246 μA/ppm. The limit of detection (LOD) was obtained from experiment at concentrations as low as 1 ppm, based on a signal-to-noise ratio (S/N) of 3, and the limit of quantization (LOQ) at 3 ppm based on a S/N of 10.

### 3.2. TLC Coupled with Colorimetric Paper-Based Analytical Device for the Separation and Detection of Ferulic Acid

#### 3.2.1. Effect of Sodium Carbonate Concentration

The colorimetric detection based Folin-Ciocalteau total phenolic assay, the commonly used method for the determination of phenolic contents was used in this study. This assay works by measuring the change in color when metal oxides are reduced. The Folin-Ciocalteau reagent reacts with phenolic compounds under basic conditions, which are obtained by adjusting the sample pH with sodium carbonate. Accordingly, the effect of the sodium carbonate concentration was evaluated for ferulic acid detection in this system, where 10% (w/v) sodium carbonate was found to give the best color response (Figure S5).

#### 3.2.2. Separation and Quantitation of Ferulic Acid

The use of the paper-based colorimetric device coupled with TLC was designed for the separation and detection of ferulic acid in the presence of ascorbic acid. Ascorbic acid is a natural antioxidant that can be present in food and cosmetics and is colorimetrically detected by Folin-Ciocalteau reagent [45]. Therefore, the separation of ascorbic acid from ferulic acid before detection is required. The separation of ascorbic acid and ferulic acid by TLC was performed with a 85:15:1 (v/v/v) chloroform: methanol: formic acid mobile phase (Figure S6), where the two analytes could be clearly separated with R_f_ values of 0.70 ± 0.01 and 0.10 ± 0.01 for ferulic acid and ascorbic acid, respectively (Table S1).

After separation, the ferulic acid containing region of the TLC was leached by ethanol onto the paper device where it reacted with the Folin-Ciocalteau reagent. The color at the reaction zone was captured by camera and converted to intensity using Adobe Photoshop. The resulting color intensity was found to be proportional to the concentration of ferulic acid (Figure S7).

#### 3.2.3. Analytical Performances for Colorimetric Detection of Ferulic Acid

**Figure 6.**
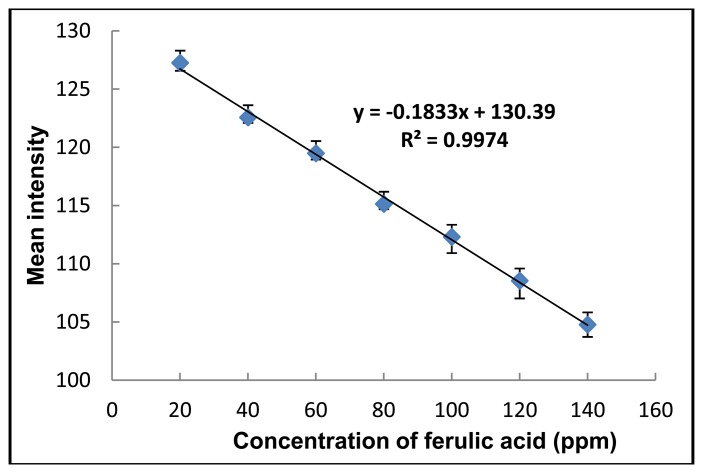
Calibration curve of ferulic acid using the paper-based colorimetric detection. Data shown are the mea ± 1 SD and are derived from 3 independent repeats.

The ferulic acid standard curve was generated by using various concentrations (1–200 ppm) of ferulic acid. The derived average color intensity from three replicate runs plotted against the ferulic acid concentration (Figure 6) revealed a linear detection range between 20 and 140 ppm (R^2^ = 0.9974), with a LOD of 7 ppm and a LOQ of 20 ppm.

### 3.3. Analytical Application in Real Samples

To verify the applicability of the proposed two paper-based analytical devices and methodology developed here, ferulic acid in real samples with a simple matrix and a complex matrix were evaluated.

#### 3.3.1. Simple Matrix Samples

The ferulic acid level of corn cider and corn milk, sample with simple matrix were determined by electrochemical detection using the standard addition method with the same experimental conditions as reported above. The two samples were each divided into two portions and then spiked with 20 and 50 ppm of ferulic acid, respectively. The recovery efficiencies, using the electrochemical detection, obtained for the samples spiked with ferulic acid revealed a recovery in the range of 96.9–99.9% and 96.9–103.2% for the corn cider and corn milk, respectively (Table 1). Samples were also analyzed by the standard HPLC-UV method so as to validate the proposed device [46]. The results obtained from those two methods were in good agreement and showed no significant differences (*p* > 0.05) between the proposed method and standard method (Table 1).

#### 3.3.2. Complex Matrix Samples

For the colorimetric detection after TLC separation, the results obtained for ferulic acid analysis in the three cosmetic samples were found to be (1) 39.76 ± 0.21 ppm; (2) 47.40 ± 0.34 ppm and (3) 31.19 ± 0.23 ppm, respectively, which compare well with that obtained by the standard HPLC-UV method of (1) 40.09 ± 0.14 ppm; (2) 48.76 ± 0.26 ppm and (3) 29.11 ± 0.16 ppm, respectively, with no significant difference (*p* > 0.05) between the proposed and standard methods.

Consequently, the two methods for the determination of ferulic acid using the paper-based analytical devices reported here are acceptable and potentially feasible for use with real samples.

## 4. Conclusions

A practical, rapid, highly sensitive and accurate method for the quantitative determination of ferulic acid in simple and complex matrices was successfully developed using a paper-based analytical device. For direct determination in a simple matrix using the paper-based electrochemical device, the DPV provided a well-defined oxidation peak of ferulic acid. The calibration curve of ferulic acid showed a good linearity in the concentration range of 3–140 ppm (R^2^ = 0.9994). The limit of detection (LOD) and the limit of quantization (LOQ) of the proposed method were found to be 1 ppm and 3 ppm, respectively. In the second design for monitoring ferulic acid in a complex matrix, the device had a LOD and LOQ for ferulic acid of 7 and 20 ppm, respectively, and was linear over the 20–140 ppm range. The benefits of the colorimetric paper-based analytical device are ease of use and analysis, rapidity, inexpensive instruments and an off-site laboratory. Furthermore, this method was successfully used for the quantification of ferulic acid in corn juice and cosmetic serums. In conclusion, these proposed paper-based analytical platforms provide a potential attractive alternative method for the determination of ferulic acid in food and cosmetic samples. As such, they may prove to be useful for nutritional and clinical investigations of ferulic acid levels in a variety of samples.

## Supplementary Material



## Figures and Tables

**Table 1.** Determination of ferulic acid levels (ppm) in real samples using the proposed paper-based device with electrochemical detection, compared to that derived using the conventional HPLC-UV. Data shown are the mean ± 1 SD derived from three independent repeats. No significant difference (*p* > 0.05) was found between the two methods in all cases.

**Samples**	**Ferulic acid (ppm ± SD, n = 3)**			**% Recovery**	**% RSD**

	Added	**Found**			

		**Proposed Method**	**HPLC-UV**		
Corn cider	0	0.24 ± 0.11	0 ± 0.00	0	7.68
	20	19.61 ± 0.16	18.96 ± 0.37	96.85	5.91
	50	50.20 ± 0.40	51.01 ± 0.53	99.92	8.92

Corn milk	0	0.94 ± 0.01	1.09 ± 0.09	0	9.19
	20	21.58 ± 0.04	20.47 ± 0.27	103.20	5.47
	50	49.41 ± 0.13	48.01 ± 0.27	96.94	8.76
