# Supplementary material for: Simple and Rapid Determination of Ferulic Acid Levels in Food and Cosmetic Samples Using Paper-Based Platforms
Source: Sensors (Basel). 2013 Sep 26;13(10):13039–53. doi: 10.3390/s131013039 (PMC3859048; doi:10.3390/s131013039)
Supplement: Supplementary file 1 [file sensors-13-13039-s001.pdf]

OPEN ACCESS

sensors

ISSN 1424-8220

www.mdpi.com/journal/sensors

Supplementary Information

Simple and Rapid Determination of Ferulic Acid Levels in Food and Cosmetic Samples Using Paper-Based Platforms. *Sensors* 2013, *13*, 13039-13053

Prinjaporn Tee-ngam [1], Namthip Nunant [2], Poomrat Rattanarat [2], Weena Siangproh [3,*] and Orawon Chailapakul [2,4,*]

1 Petrochemistry and Polymer Science, Faculty of Science, Chulalongkorn University, Patumwan, Bangkok 10330, Thailand; E-Mail: jui_jitzu14@hotmail.com

2 Electrochemistry and Optical Spectroscopy Research Unit, Department of Chemistry, Faculty of science, Chulalongkorn University, Patumwan, Bangkok 10330, Thailand; E-Mails: nongpang_2527@hotmail.com (N.N.); r.poomrat@hotmail.com (P.R.)

3 Department of Chemistry, Faculty of Science, Srinakharinwirot University, Sukhumvit 23, Wattanna, Bangkok, 10110, Thailand

4 National Center of Excellence for Petroleum, Petrochemicals, and Advanced Materials, Chulalongkorn University, Bangkok 10330, Thailand

* Authors to whom correspondence should be addressed; E-Mails: weenasi@hotmail.com (W.S.); corawon@chula.ac.th (O.C.); Tel.: +66-2-649-5000 (ext. 8208) (W.S.); +66-2-218-7615 (O.C.); Fax: +66-2-259-2097 (W.S.) ; +66-2-218-7615 (O.C.).

Figure S1. Schematic of representation of photolithography and screen printing for paper-based electrochemical device.

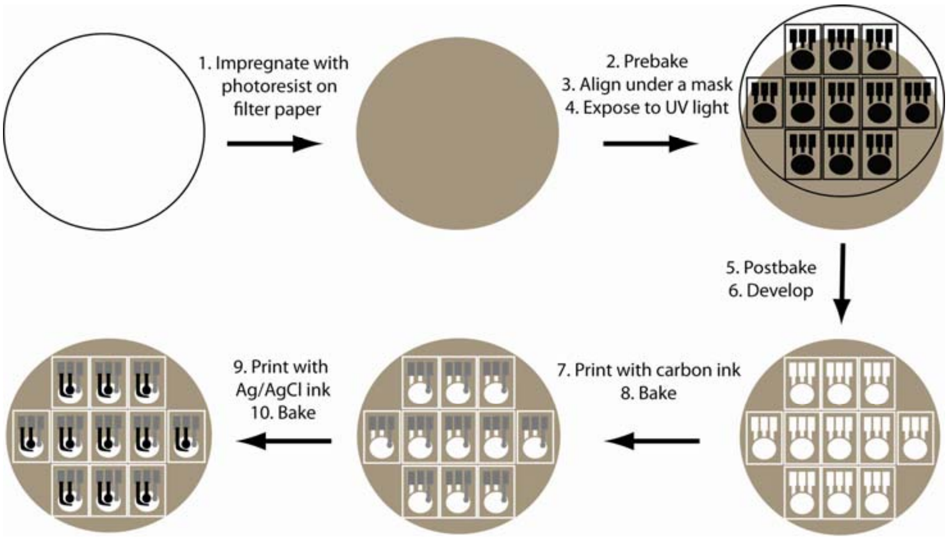

Figure S2. Schematic of representation of melting wax screen printing for colorimetric paper-based analytical device.

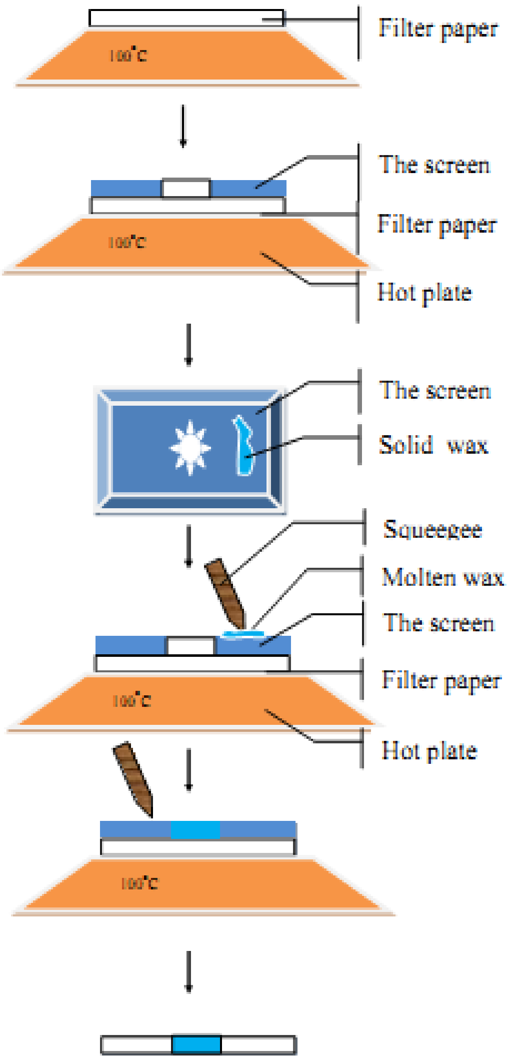

Figure S3. The influence of scan rate on the oxidation peak currents of 50 ppm ferulicacid in 0.1 mol L^{-1}HAc- NaAc buffer solution.

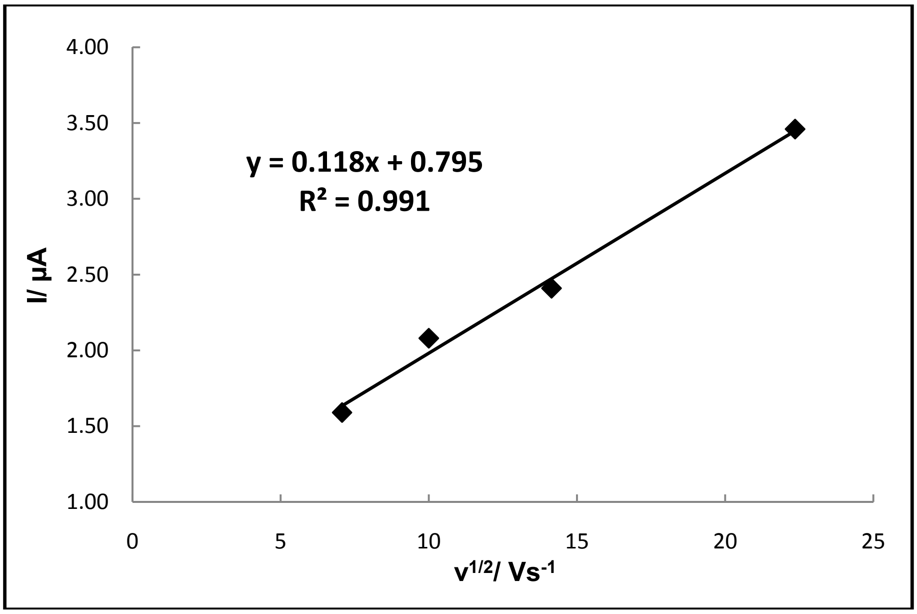

Figure S4. Differential pulse voltammograms of 50 ppm ferulic acid in 0.1 mol L^{-1} HAc-NaAc buffer solution (pH 5) at different pulse amplitude.

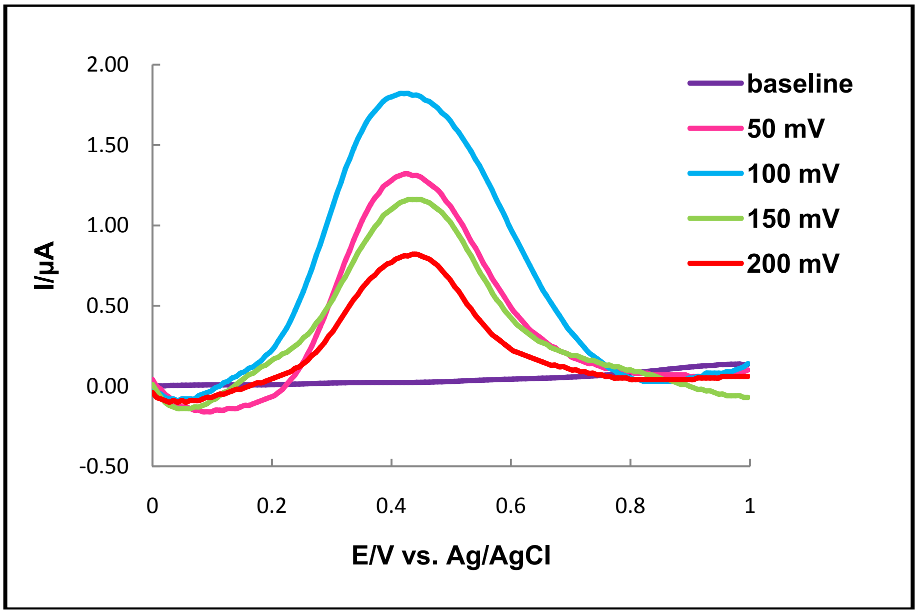

Figure S5. The effect of concentration of sodium carbonate on mean intensity.

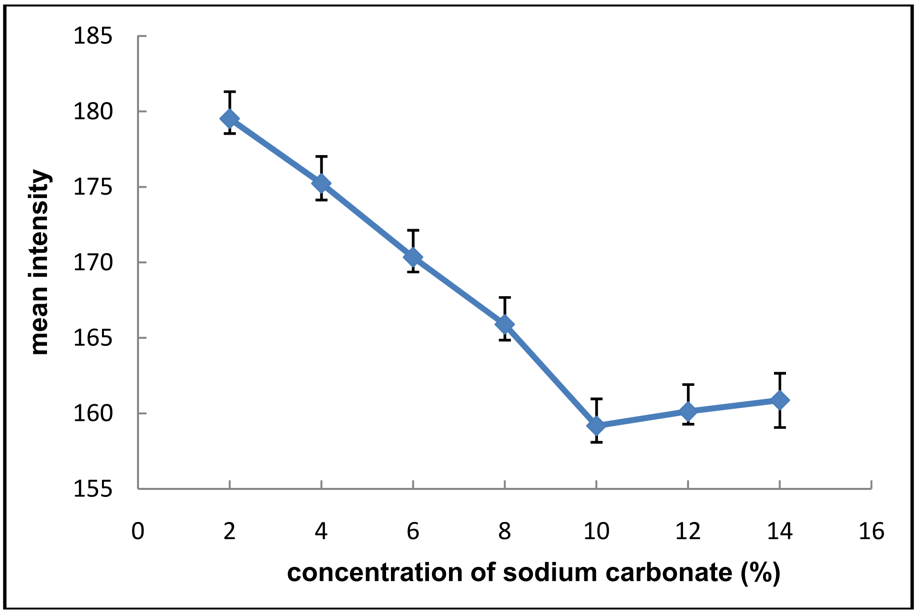

Figure S6. Separation of ferulic acid and ascorbic acid by thin-layer chromatography (**a**) Spot of ferulic acid; (**b**) Spot of ascorbic acid; (**c**) Spot of ferulic acid and ascorbic acid.

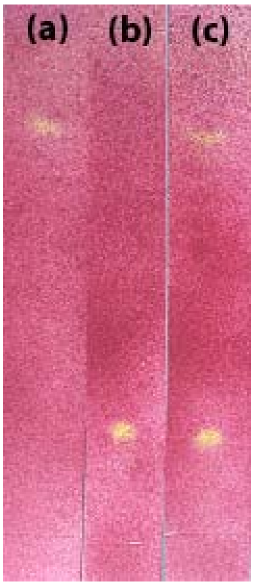

Figure S7. Thin- layer chromatography couple with colorimertric paper-based analytical device. Intensity of the observed color is proportional to the concentration of ferulic acid.

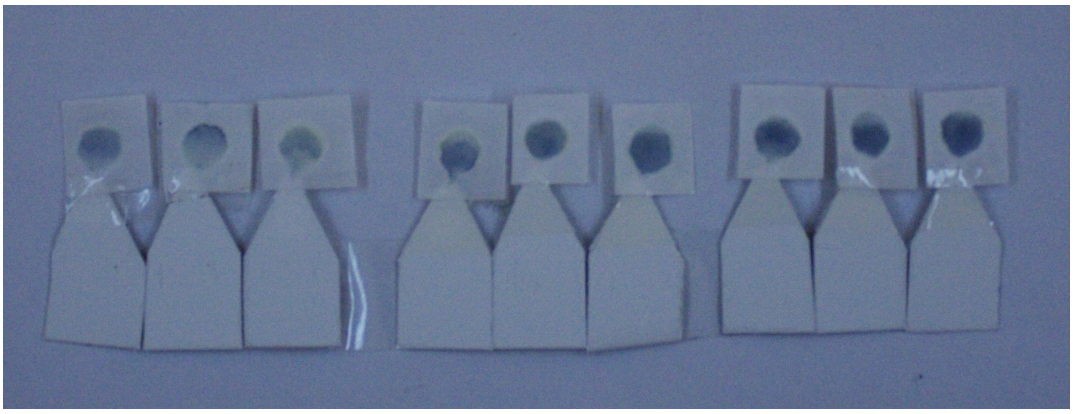

Table S1. R_f value of ferulic acid and ascorbic acid in solvent system chloroform: methanol: formic acid (85: 15: 1).

		**R_f value**	**Average R_f**
	1	0.72	
Ferulic acid	2	0.70	0.70 ± 0.01
	3	0.71	
	1	0.10	
Ascorbic acid	2	0.10	0.10 ± 0.00
	3	0.10	

© 2013 by the authors; licensee MDPI, Basel, Switzerland. This article is an open access article distributed under the terms and conditions of the Creative Commons Attribution license (http://creativecommons.org/licenses/by/3.0/).
